# Colour plasticity in the shells and pearls of animal graft model *Pinctada margaritifera* assessed by HSV colour quantification

**DOI:** 10.1038/s41598-019-43777-4

**Published:** 2019-05-17

**Authors:** Pierre-Louis Stenger, Jérémie Vidal-Dupiol, Céline Reisser, Serge Planes, Chin-Long Ky

**Affiliations:** 1 IFREMER, UMR 241 Écosystèmes Insulaires Océaniens, Labex Corail, Centre Ifremer du Pacifique, BP 49, 98725 Tahiti, French Polynesia; 2 IFREMER, UMR 5244 IHPE, University Perpignan Via Domitia, CNRS, University Montpellier, F-34095 Montpellier, France; 3 PSL Research University, EPHE-UPVD-CNRS, USR 3278 CRIOBE, Labex Corail, Université de Perpignan, 52 Avenue Paul Alduy, 66860 Perpignan Cedex, France

**Keywords:** Imaging, Zoology

## Abstract

The bivalve *Pinctada margaritifera* has the capacity to produce the most varied and colourful pearls in the world. Colour expression in the inner shell is under combined genetic and environmental control and is correlated with the colour of pearls produced when the same individual is used as a graft donor. One major limitation when studying colour phenotypes is grader subjectivity, which leads to inconsistent colour qualification and quantification. Through the use of HSV (Hue Saturation Value) colour space, we created an R package named ‘*ImaginR*’ to characterise inner shell colour variations in *P*. *margaritifera*. Using a machine-learning protocol with a training dataset, *ImaginR* was able to reassign individual oysters and pearls to predefined human-based phenotype categories. We then tested the package on samples obtained in an experiment testing the effects of donor conditioning depth on the colour of the donor inner shell and colour of the pearls harvested from recipients following grafting and 20 months of culture *in situ*. These analyses successfully detected donor shell colour modifications due to depth-related plasticity and the maintenance of these modifications through to the harvested pearls. Besides its potential interest for standardization in the pearl industry, this new method is relevant to other research projects using biological models.

## Introduction

The marvellous diversity of colours among molluscan shells has been widely esteemed for hundreds of years by collectors and scientists^1^. Colour is a well-known naturally plastic trait that varies due to inter-individual genetic differences, crystalline structure of exoskeletons or appendages, variable diet or other environmental conditions^1–6^. Colour phenotype has been the centre of interest in many biological studies since it is involved in some evolutionary processes (natural selection for camouflage or defence, sexual selection for fitness in a partner, etc.) and can be exploited for industrial purposes (dyes, jewellery)^1,7–10^. For the pearl industry, this trait has been selected to create commercially specific strains such as golden Pacific oysters *Magallana gigas* (Thunberg, 1793)^11–13^, “ivory” strains of the Japanese scallop *Patinopecten yessoensis* (Jay, 1987)^6,14^ and “gold” pearl production from the silver- or gold-lipped pearl oyster *Pinctada maxima* (Jameson, 1901)^15^. The black-lipped pearl oyster, *Pinctada margaritifera* (Linnaeus, 1758), is the top aquaculture species in French Polynesia. The species is cultivated for pearl production and the associated industry is the second greatest source of revenue for French Polynesia after tourism^16^. *P*. *margaritifera* has the ability to produce cultured pearls with a very wide range of colours^17,18^, derived from the donor oysters used to provide grafting tissue^19–22^. The pearl production process depends upon a grafting operation requiring a donor pearl oyster, a recipient pearl oyster and a skilled technician to perform the surgery^23^. A piece of tissue from the edge of the mantle of a donor oyster (the “saibo”, also known as the graft), selected based on the quality of the donor’s shell colour, is inserted together with a nucleus (made of mollusc shell or synthetic material) into a recipient oyster chosen for its vigor^23,24^. The external shell of *P*. *margaritifera* is commonly black, sometimes with white dotted lines arranged in lateral bands. However, some individuals are red in colour over the entire surface of their shells^18^, while others are yellow just at the base of the external shell surface. A very rare white albino shell morphotype (named “pupure”) also exists^25^. The inner side of the shell has a larger colour gamut, ranging from red to yellow, green to blue or peacock to white, with all possible intermediate nuances^21,26^. Pigments are primarily visible on the peripheral parts of the shell^27^. Indeed, because the nacreous layer covers the prismatic layer, this may make the pigments opaque^27–29^. According to Ky *et al*.^19^, the factors contributing to the colour of a pearl include the phenotype of the donor oyster (partial genetic inheritance), geographical location, and the environmental conditions in which the recipient oyster is reared during pearl development^30^.

**Figure 1:**
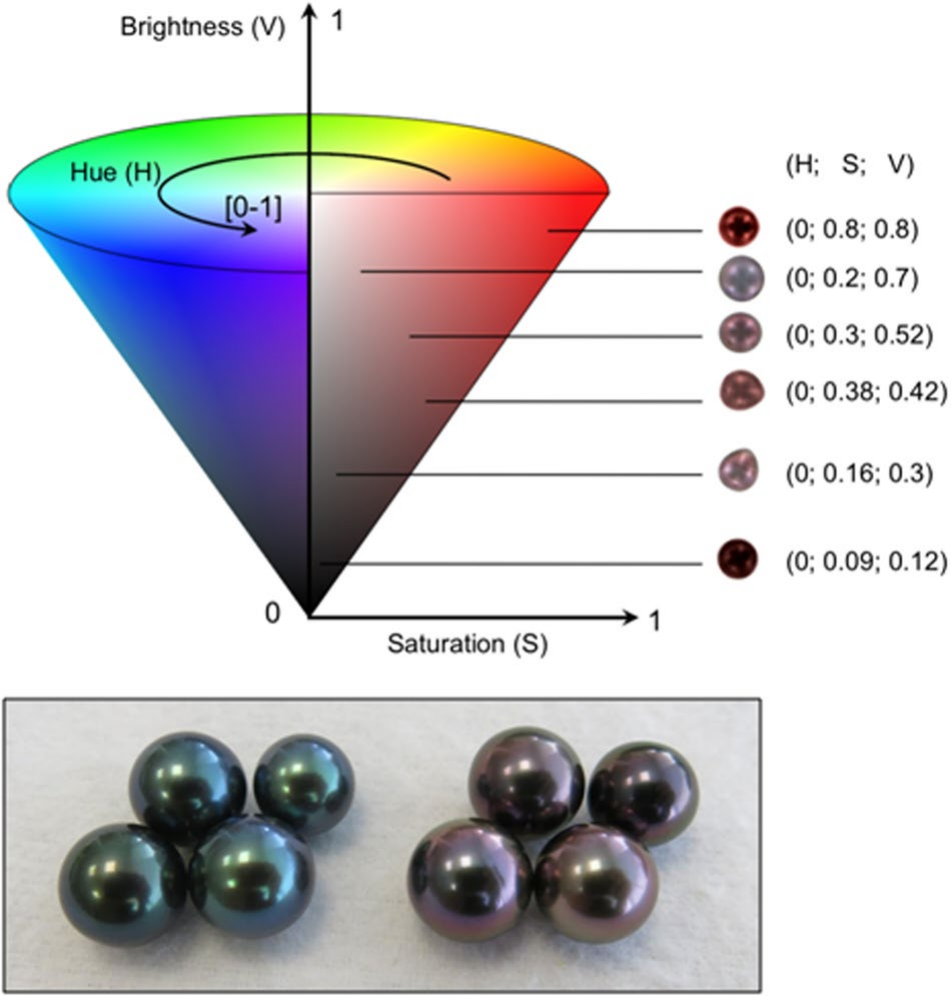
The Hue Saturation Value (HSV) colour code system used to quantify colour in *Pinctada margaritifera* shells and cultured pearls. For a determined hue (H), saturation (S) range (from 0, white, to 1), and brightness (V) range (from 0, black, to 1) were measured, as illustrated here by red pearl samples, with HSV values shown in brackets. Red and green pearl samples are illustrated above the conical representation of the HSV system.

The assessment of colour nonetheless remains a subjective trait in which human quantification and qualification can be strongly biased, as visual perception of colours differs between individuals^31^. For this reason, efforts are currently being made to develop accurate and reproducible computational methods for automatic objective colour qualification and quantification^10^. In this study, based our approach on the HSV (Hue Saturation Value) colour space to characterize the colour variation in our biological model (Fig. 1).

The objective of the present study is the qualification and quantification of colour with a new standardized and reproducible method. We used HSV colour space to characterise colour variation in the inner shell of black-lipped oysters and pearls produced by the pearl industry. Finally, to enable a wider use of this method in scientific and private programmes, we developed an R package, *ImaginR*^32^ and made this publicly available.

## Results

### Shell and pearl materials issued from an experimental graft

To validate our newly developed method for qualifying and quantifying colours, we first used material issued from a basic experimental graft on which we assessed the colour of donors and of pearls produced with the grafts of these donors. For this graft, the mantle (the biomineralizing tissue) from each of the donors selected for their colours was cut into 30 pieces, which were then grafted into the gonads of 30 recipient oysters in order to produce pearls.

Colours were then analysed in a total of 669 pearls issued from grafts made with two donor colour phenotypes (green and red) and cultured at two depths (4 or 30 m) to create a baseline for measuring colour determination stability using the new colour assessment method. The average nucleus retention rate of the experimental graft was 93.0% (N = 749) at 42 days post-grafting. After 20 months of culture, pearls were successfully harvested from 89.9% of the individuals initially grafted (N = 669). The difference (3.1%) corresponded to nucleus rejection after day 42, and oyster mortalities. The numbers of pearls harvested per donor colour class and per depth group were: 363 pearls formed by grafts from the green donor phenotype (197 for the group at 4 m and 166 for the group at 30 m) and 306 pearls formed by grafts from the red donor phenotype (143 for the group at 4 m and 163 for the group at 30 m).

### Hue values for inner shells and cultured pearls

**Figure 2:**
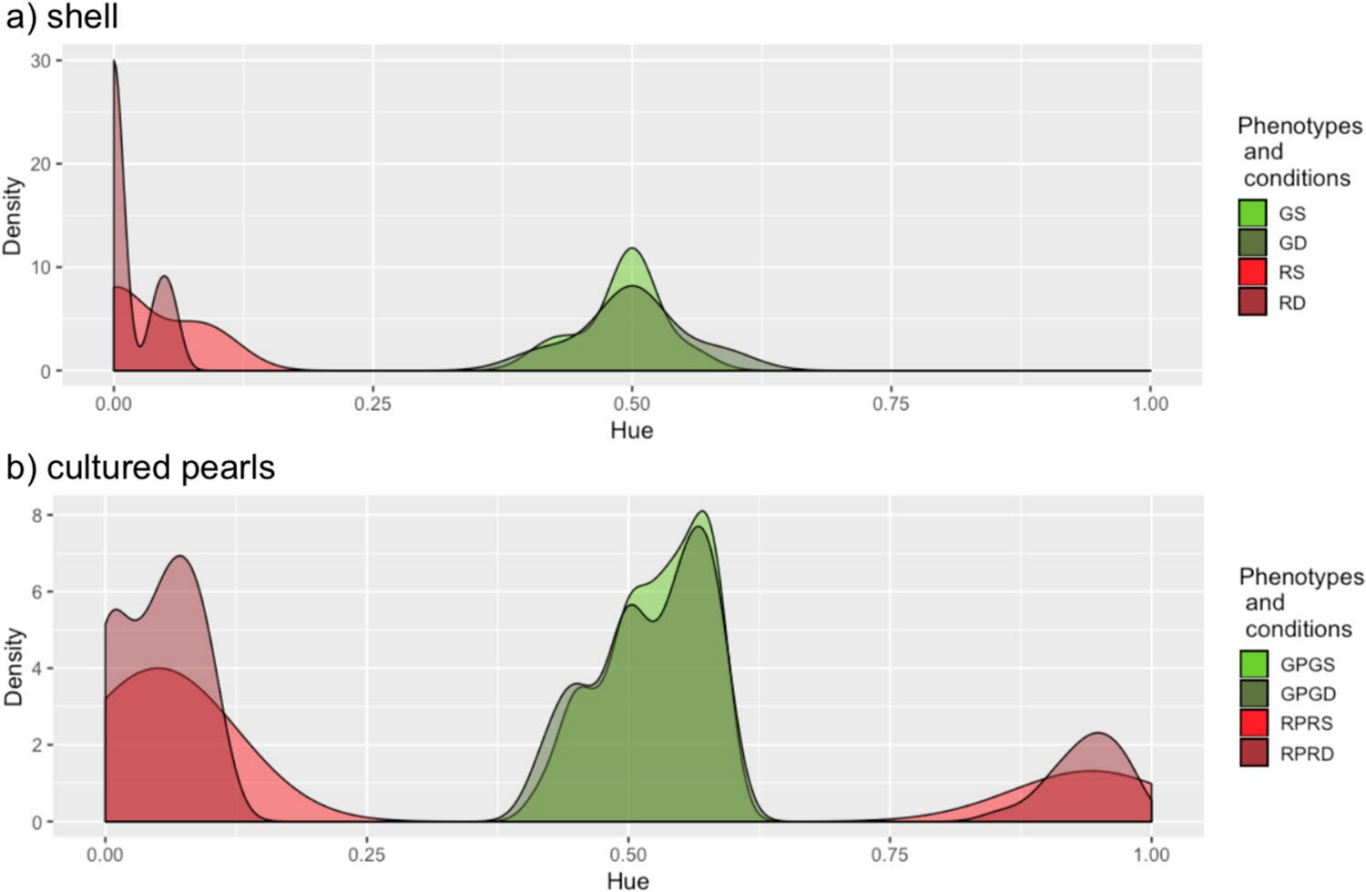
Hue density plot distribution of *P*. *margaritifera* for: (**a**) donor oyster shells from: GS (green donors reared at 4 m; N = 9), GD (green donors reared at 30 m; N = 6); RS (red donors reared at 4 m; N = 5), and RD (red donors reared at 30 m; N = 7), (**b**) cultured pearls associated with the donors illustrated in (**a**), with GPGS (green pearls from green donors reared at 4 m; N = 132), GPGD (green pearls from green donors reared at 30 m; N = 66), RPRS (red pearls from red donors reared at 4 m; N = 84) and RPRD (red pearls from red donors reared at 30 m; N = 118). Light green (or red) and dark green (or red) distributions correspond to donor conditioning in subsurface (4 m) or deep (30 m) culture, respectively, prior to graft operations.

The hue distributions for the shell phenotypes and rearing conditions revealed four dominant hues for green donors reared at 4 m depth (GS) (0.500; 0.555; 0.444; 0.4166), three for green donors reared at 30 m (GD) (0.500; 0.583; 0.416), three for red donors reared at 4 m (RS) (0.000; 0.066; 0.100) and three for red donors reared at 30 m (RD) (0.000; 0.055; 0.04) (Fig. 2a).

Overall, 138 different hues were found among the 363 pearls produced with grafts from green donors and 185 hues for the 306 pearls produced with grafts from red donors (Table 1). Some of the pearls produced with grafts from green donors reared at 4 m (PGDS) and 30 m (PGDD) shared the same hues, as the diversity index (ratio of the hue number and pearl number) for PGD total (0.38) was lower than both the PGDS (0.43) and PGDD (0.59) diversity indices. The hue diversity index was greater for the red phenotype (0.60 in total) than the green, even though the red donor phenotype also shared hues between pearls from grafts of the donors conditioned at 4 m (PRDS) (0.72) and pearls from grafts of the donors conditioned at 30 m (PRDD) (0.69) (Table 1). Statistically, we observed more green pearls from the grafts of green donors when these donors had been reared at 4 m than at 30 m (67% at 4 m and 39.6% at 30 m, Chi^2^ test *p* < 0.001) (Table 1). However we observed the opposite phenomenon for the red phenotype. Indeed, we observed more red pearls from the graft of red donors when these had been reared at 30 m than at 4 m (58.7% at 4 m and 72.4% at 30 m, Chi^2^ test *p* < 0.05) (Table 1). Among the pearls produced with grafts from donors grown at 4 m, there were more green pearls from the green donor grafts (67%) than red pearls from the red donor grafts (58.7%). At 30 m, the opposite phenomenon was shown, with more red pearls from the red donor grafts (72.4%) than green pearls from the green donor grafts (39.6%) (Chi^2^ test *p* < 0.001). When the pearls from the two depths were considered together, the same pattern was found overall, with again more red pearls from red donors than green pearls from green ones (65.55% for red and 53.3% for green; Chi^2^ test *p* < 0.005; Table 1).

**Table 1:** Number of pearls and pearl hues categorized by phenotype and culture treatment.

Donor conditioning depth	Green donors			Red donors		
	4 m	30 m	Total	4 m	30 m	Total
Number of pearls	197	166	363	143	163	306
Number of green (or red) pearls from green (or red) donors	132	66	198	84	118	202
Rate (%) of green (or red) pearls from green (or red) donors	67.0	39.6	53.3	58.7	72.4	65.55
Number of hues	84	98	138	103	112	185
Diversity index	0.43	0.59	0.38	0.72	0.69	0.60

The percentages (line number 4) represent the proportion of pearls with same colour phenotype as their donors. Comparisons of this rate were all significant: 1) between conditioning depth (4 m vs. 30 m), within green (*p* < 0.001) and red donors (*p* < 0.05); and 2) between green and red donors for the 30 m conditioning depth (*p* < 0.001) and for both depths together (*p* < 0.005). All *p-*values were obtained from Pearson’s Chi-squared test with Yates continuity correction test. The diversity index was obtained by calculating the ratio of the number of hues to the number of pearls.

### The analysis of saturation for inner shell colour and cultured pearls

**Figure 3:**
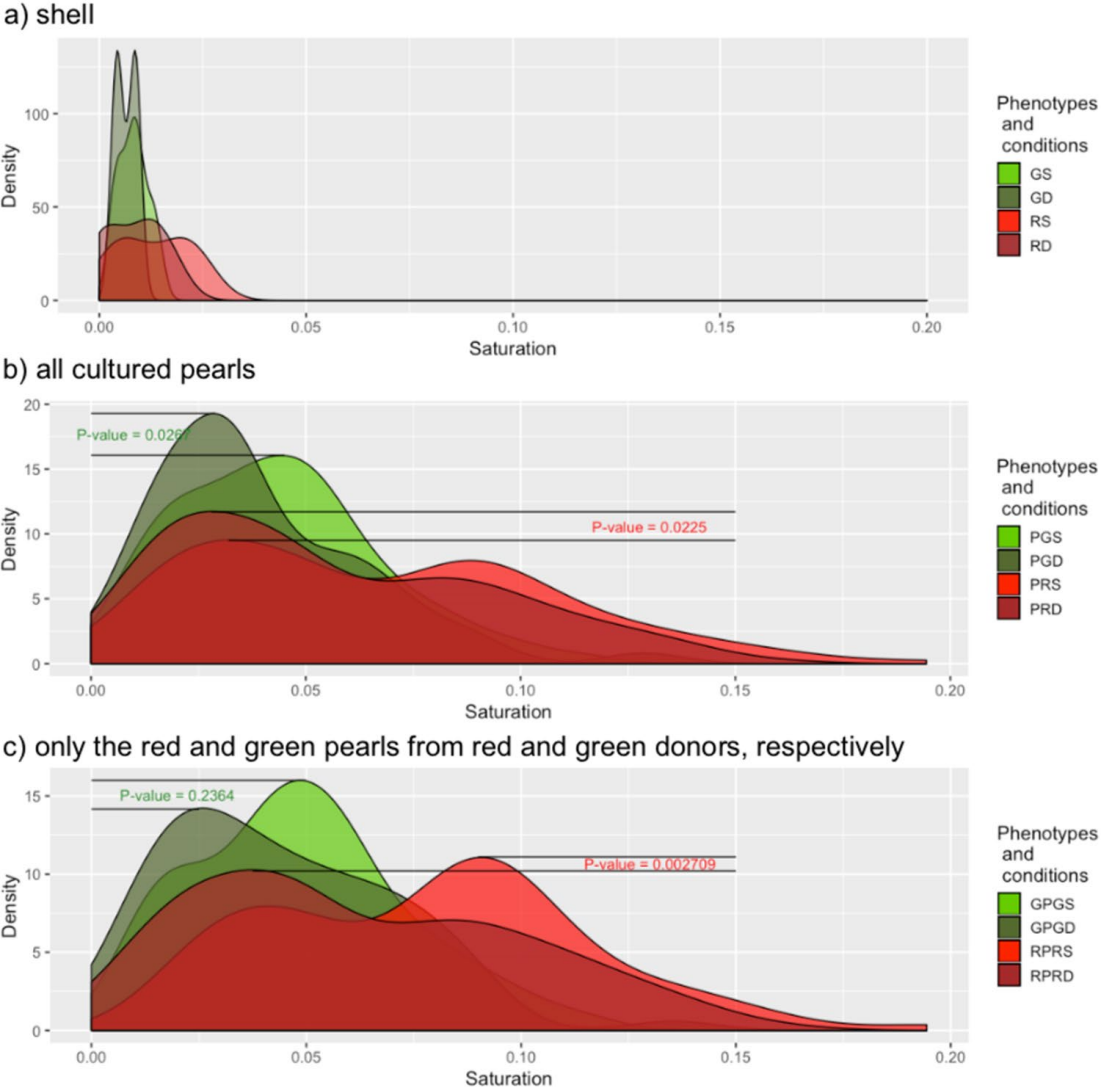
Saturation density plot distribution of *P*. *margaritifera* for: (**a**) shells of donor oysters from: GS (green donors reared at 4 m; N = 9), GD (green donors reared at depth of 30 m; N = 6), RS (red donors reared at 4 m; N = 5), and RD (red donors reared at 30 m; N = 7), (**b**) multicoloured cultured pearls associated with the donors in (**a**), with PGDS (pearls from green donors reared at 4 m; N = 197) and PGDD (pearls from green donors reared at 30 m; N = 166); PRDS (pearls from red donors reared at 4 m; N = 143) and PRDD (pearls from red donors reared at 30 m; N = 163), (**c**) associated cultured pearls that show the same colour hue as their corresponding donors, GPGS (green pearls from green donors reared at 4 m; N = 132), GPGD (green pearls from green donors reared at 30 m; N = 66), RPRS (red pearls from red donors reared at 4 m; N = 84), and RPRD (red pearls from red donors reared at 30 m; N = 118). Light green (or red) and dark green (or red) distributions correspond to donor conditioning in subsurface (4 m) or deep (30 m) culture, respectively, prior to graft operations.

The distributions of donor inner shell colour shifted closer towards low saturation for the 30 m depth group than for the 4 m group (Fig. 3a).

When all cultured pearls were considered, samples from both green (*p* < 0.05) and red (*p* < 0.05) donors showed a significant shift of saturation towards lower levels following deep conditioning (30 m) compared with sub-surface (4 m) conditioning (Fig. 3b).

When only red pearls from grafts of red donors and green pearls from grafts of green donors were considered, the saturation distribution also significantly shifted towards lower saturation, with greater depth for RPRD only (*p* < 0.005 and *p* = 0.2364, respectively). These results are similar when all cultured pearls were considered, but the differences between the depths were 140.8 times stronger for the red phenotype (Fig. 3c). In terms of saturation, the inner shell colour therefore became less intense and less bright with depth.

### Brightness (V) of donor oyster inner shells and cultured pearls

**Figure 4:**
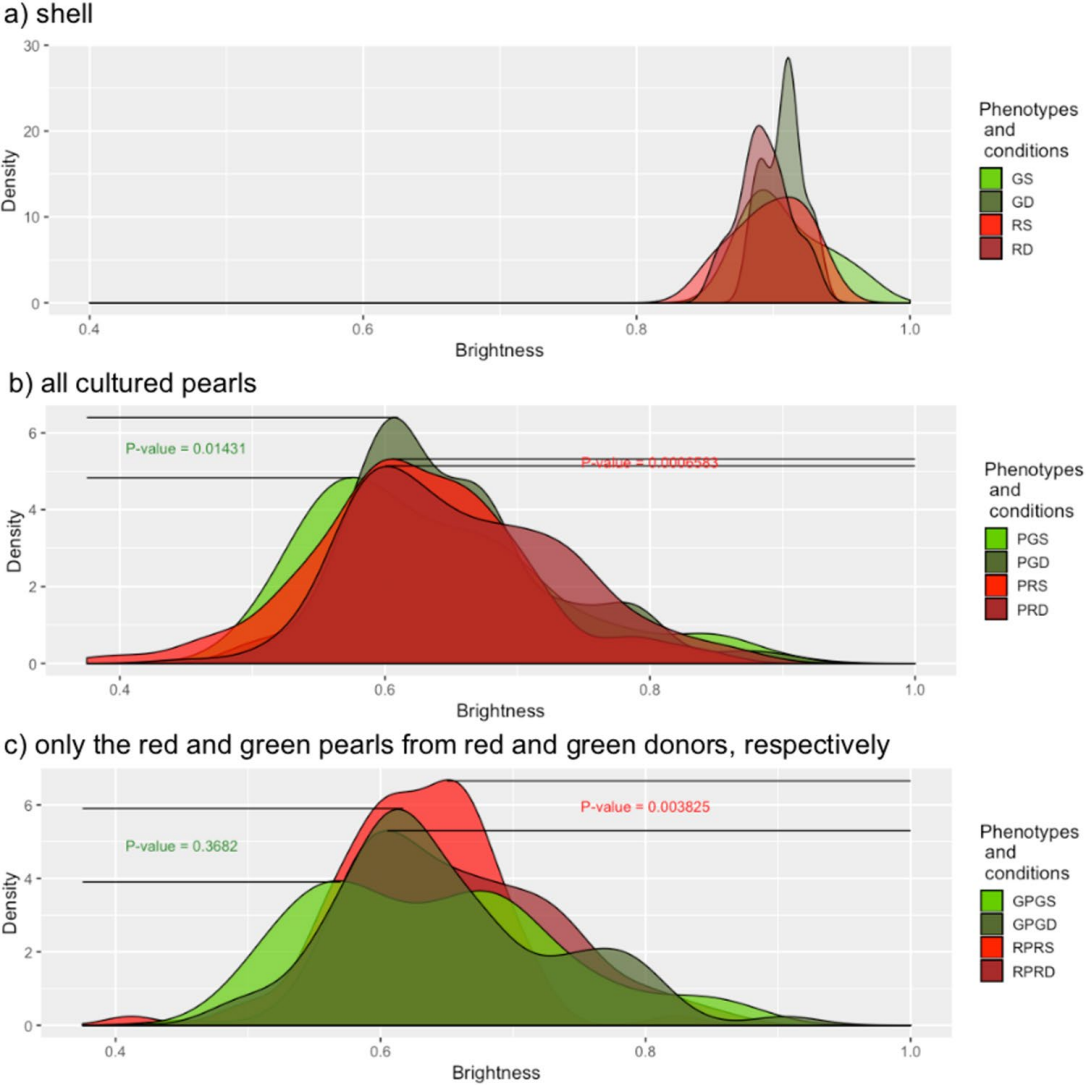
Brightness density plot distribution of *P*. *margaritifera* for: (**a**) shells of donor oysters from GS (green donors reared at 4 m; N = 9), GD (green donors reared at 30 m; N = 6), RS (red donors reared at 4 m; N = 5), and RD (red donors reared at 30 m; N = 7), (**b**) associated cultured pearls that can have different colours, PGDS (pearls from green donors reared at 4 m; N = 197), PGDD (pearls from green donors reared at 30 m; N = 166), PRD4 (pearls from red donors reared at 4 m; N = 143), and PPDD (pearls from red donors reared at 30 m; N = 163), and (**c**) associated cultured pearls that have the same colour hue as their donors, GPGS (green pearls from green donors reared at 4 m; N = 132), GPGD (green pearls from green donors reared at 30 m; N = 66), RPRS (red pearls from red donors reared at 4 m; N = 84), and RPRD (red pearls from red donors reared at 30 m; N = 118). Light green (or red) and dark green (or red) distributions correspond respectively to donor conditioning in subsurface (4 m depth) or depth culture (30 m depth), prior to graft operations.

Regarding the inner shell colour, the brightness (V) of the distributions shifted to higher levels in the samples conditioned at 30 m depth compared with those conditioned at 4 m depth (Fig. 4a).

When all cultured pearls were considered, samples from both green (*p* < 0.001) and red (*p* = 0.014) donors had a significant shift to higher values of V following the deeper conditioning (30 m) compared with sub-surface (4 m) (Fig. 4b).

When red pearls from grafts of red donors (*p* < 0.005) and green pearls from grafts of green donors (*p* = 0.3682) were considered separately, the distribution of V values shifted significantly to greater darkness with depth, but this difference was not significant for the green pearls (Fig. 4c). The differences between the depths were 5.07 times greater for the red phenotype considered alone. In terms of darkness, the inner shell colour became more grey, dull and drab at greater conditioning depth.

## Discussion

Our R package, based on image analysis with HSV colour space and machine-learning approaches, validated the method used through the analysis of the influence of depth on the colour of two economically important pearl donor phenotypes: the green and the red inner shell phenotypes. Our results show that (i) the R package successfully categorized the pearl phenotypes used in this study, that (ii) cultivation environment of the donor oysters (here depth) heavily influences the brightness V and the saturation S of the colour, and (iii) that the colour variation related to depth was transmitted from donors to pearls. The *ImaginR* package is thus suitable for indicating colour variations in oysters and could now be used in other biological models of animal or plant origin. The package was deposited in CRAN under the name *ImaginR* V2.0 (https://cran.r-project.org/web/packages/ImaginR/index.html)^32^.

We developed this tool in the form of an R package to reduce the subjectivity in quantifying and qualifying colour in the pearl oyster *P*. *margaritifera*. Marchais *et al*.^33^ were the first to develop a new method based on digital colour analysis, using HSL colour space, to highlight the link between shell colour and algal pigments under experimental conditions. However, the HSL colour space has an unfortunate interaction between brightness and saturation during image processing^34^. Thus, for a maximum lightness value, with HSL, the saturation always gives white data, while this problem does not arise with HSV colour space^34^, which gives values closer to human vision^35,36^. Trinkler *et al*.^37^ already attempted to measure and quantify colour in juvenile *P*. *margaritifera* with an International Commission on illumination (ISI) chromaticity diagram, but did not find any differences of colour trend in the shells. We therefore decided to create our own R package adapted to our biological model (*P*. *maragaritifera*), using HSV colour space. To our knowledge, there is no other free software with these characteristics.

Interestingly, we observed that the differences induced by depth in the red donor phenotype were higher overall than those in the green phenotype. This could provide some first clues on how colour is genetically controlled in *P*. *margaritifera*. Indeed, we could hypothesize that by being more “stable” in terms of colouration change, the green phenotype could be controlled by more genes than the red phenotype. Multifactorial genetic control of a trait is often synonymous with a continuous phenotype, in which variations might be more subtle^38,39^. This could mean that the molecular pathways leading to the expression of the colour of these two phenotypes differ. It would be interesting to work towards a better understanding of the molecular control of expression of the colour phenotypes of *P*. *margaritifera*, to see if our suppositions are correct.

The effects of depth on the colouration of marine animals have been little described so far, but there are nonetheless some reports^40–42^. Change in colour correlated with depth can be explained by many other environmental factors linked to depth, such as diet composition^40,43^, temperature^44^, light levels^42^, or even pressure^45^, which complicates its study. Southgate & Lucas^41^ report absolutely no influence of hydrostatic pressure on *P*. *margaritifera*, but other environmental parameters associated with depth are known to have significant influences. Light decreases with depth and, according to Gervis and Sims^42^, so does pearl quality and colour. Indeed, *Pinctada fucata martensii* (Gould, 1850) produces high quality pinkish pearls below 5 metres, but nacre deposition is maximized under blue light like that found in deeper water. Food intake can also influence nacre deposition. Indeed, according to Joubert *et al*.^43^, when trophic levels are high (microalgal concentration), there is a decrease in aragonite tablet thickness, but a strong increase in the speed of nacreous deposit. However, Latchère *et al*.^46^ demonstrated that food level had no effects on quality traits of *P*. *margaritifera* mineralization. It is also well known that water temperature strongly influences bivalve metabolism and physiological processes^47–49^. Indeed, temperature influences the relative expression of genes involved in biomineralization^43,46^. So there can me multiple environmental reasons for such changes. Furthermore, Rousseau & Rollion-Bard^45^ found variation in the shape of nacre tablets as a function of depth, in relation to the shell growth direction. The shape of the tablets changed from hexagonal to rhomboid at a depth of 39 m. With this modification in shape, the tablets become larger, but also thinner, so that the new pigments laid down are more visible by transparency. According to these authors, the iridescent colours are affected by the thickness of the layers. Pigment concentration also depends on their final location in the shell layers^43,46^. The shell of the black-lipped pearl oyster has four layers, from exterior to interior: (i) the periostracum, (ii) the prismatic layer, (iii) the fibrous layer, and (iv) the nacreous layer^28,50^. These layers show progressive changes in their crystalline structure and associated organic matrix structure^28^. The precise location, kinds of pigments and the relative quantities of these pigments in each of these different layers are not yet well known.

Our results indicate that depth can change the expression of colour and it was already well known that the environment can influence the expression of biomineralizing genes^43,46^. We hypothesize that a thickness gradient of the aragonite layer could explain why colour pigmentation only appears at the top of the inner shell. Indeed, a dorso-ventral section of a valve of *P*. *margaritifera* shows that the nacreous layer is thinner closer to the ventral side than to the dorsal side^43^ a phenomenon that would enable the pigments to be more visible here due to higher transparency in this part of the shell. As the aragonite tablets elongate faster with depth^45^, this layer could therefore act to slightly darken the pigments at the top of the inner shell.

Donor oysters conditioned at 30 m depth were used as donors to produce pearls, as were counterparts conditioned at 4 m. Interestingly, the pearls produced and harvested 20 months after grafting displayed colour variations corresponding to those expressed by the donors. The maintenance of this phenotype expression by the donor tissues suggests epigenetic regulation of the colour intensity. Indeed, environmental changes can modify individual phenotypes^51,52^ and such modifications can persist in a transplanted organ grafted into another individual^53,54^. So the graft has a memory, but we do not yet know how reversible this (memory of these modifications) is and over how much time. It would be interesting to study this level of regulation in the expression of colour in *P*. *margaritifera*.

Our results on the impact of depth on the colour of *P*. *margaritifera*, although they do not provide a description of the mechanisms, is of great importance for the Polynesian pearl industry that is still recovering from the 2008 crisis, when pearl price per gram plummeted. To ensure a sustainable future for the industry, there is a growing interest in producing fewer pearls, of better quality^23^, particularly pearls of more contrasting colours. Indeed, pearls presenting a colour with high saturation and less darkness are of great value on most markets. However, in past years, spat collection has become increasingly unreliable, and problems such as vandalism (or theft of grafted and non-grafted oysters in marine concession areas) have increased. In addition, the increase in the length and number of exceptionally warm climatic conditions (lagoon surface waters over 30 °C) has forced producers to install their lines at 30 m depth to avoid theft, or mortality due to high temperatures. In the light of our findings this could be problematic if non-grafted oysters stocked at 30 meters were subsequently used as donors. Indeed, we showed that the inner shell colour and associated pearls from donors that had spent one month at 30 m depth were darker and duller than those from source-cultivated oysters, despite the fact that, during the post-graft culture period, the recipient oysters were cultured at 4 m depth for 20 months. It would be interesting to do other similar experiments, but with more depths tested (e.g. 4 m, 10 m, 20 m and 30 m) in order to see the behaviour of colour expression variation at these different depths.

Our exploration of colour plasticity in response to an environmental difference was made with a new tool using HSV colour space. The emergence of this type of analysis could be useful not only for the pearl industry, but also in other domains and for other biological models. This method was not only able to confirm and characterize variation in the colouration of black-lipped pearl oysters but also the persistence of this phenotypic difference in pearls harvested 20 months after exposure.

## Materials and Methods

An experimental graft was performed in 2013 with two colour phenotypes of *P*. *margaritifera* donors (red and green) reared at 4 and 30 m depth. Visually, colour differences were observed in both green and red donors between the two depth groups. These four groups were used as donors in an experimental graft. After harvesting, colour differences were again found visually in the different groups of pearls. We decided to analyse these variations using a suitable colour space in an automated manner.

### Animal conditioning and experimental graft

**Figure 5:**
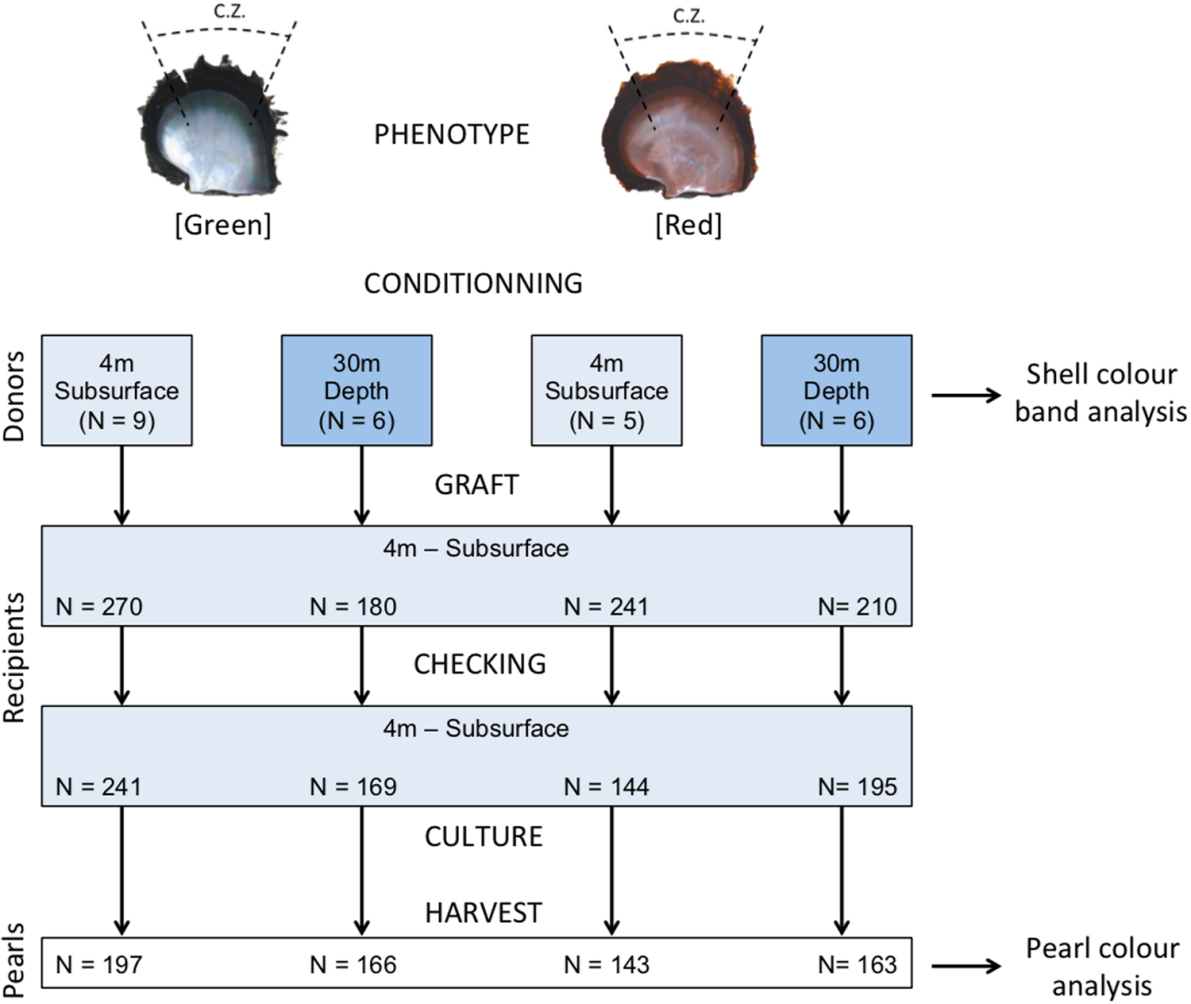
Experimental design of the experimental *Pinctada margaritifera* grafting procedure using two donor oyster phenotypes, green and red. Each phenotype was conditioned for one month prior to the graft operation, either in subsurface (4 m) or deep culture (30 m). The commercial zone (C.Z.), indicated by the dotted lines, is the section of the donor mantle from which grafts are usually cut. Thirty grafts were made from each donor. A check for nucleus retention was made 42 days post-graft and the pearls were harvested 20 months post-graft. Numbers in brackets correspond to the frequencies of donors, recipients or pearls.

Two *P*. *margaritifera* phenotypes, with either a green or a red inner shell, were selected as donors for an experimental graft (Fig. 5). The green individuals originated from Mangareva Island lagoon (Gambier archipelago, French Polynesia) and the red phenotype came from the Takaroa atoll (Tuamotu archipelago, French Polynesia). The red oysters were transferred by plane to Mangareva one month before grafting to allow acclimation. After a month, future donors of each phenotype were separated into two groups. One group was then reared at 4 m depth (N = 9 for green; N = 5 for red) and the other at 30 m depth (N = 6 for green; N = 7 for red) for one additional month in order to obtain a colour change (final donors). After the second month, the oysters were collected and used in the experimental graft operation. *P*. *margaritifera* of about two years were used to serve as recipients were collected as spat in the Mangareva Island lagoon (Gambier Archipelago, French Polynesia). Passive spat catching techniques were used with commercial collectors.

The experimental graft using these donors was performed in Regahiga Pearl Farm (Gambier archipelago, French Polynesia) in December 2013 (Fig. 5). For this graft, the mantle (the biomineralizing tissue) from donors selected for their colours (i.e. red and green) were cut into 30 small pieces known as “saibo”. These pieces of mantle were grafted into 30 recipient oysters together with a nucleus (nucleus weight: 0.2337 g; nucleus diameter: 0.56425 mm). Cells of the “saibo” (from the donor) produce nacre that builds up on the nucleus to form a pearl in the recipient oyster. Recipient and donor oysters measured approximately 12 cm in height from the bottom to the top of their shells. The shells of the donor oysters were kept for chromatic analysis.

All recipient oysters were individually labelled (with numbered colour coded plastic labels) so as to maintain traceability between donor identity, and corresponding harvested pearls. All the grafted recipient oysters were then cultured at 4 m depth. As is normal aquaculture practice, the oysters were regularly cleaned in order to remove biofouling (epibiota), which can hinder healthy oyster growth and pearl production. After 42 days, nucleus retention was checked. Finally, the pearls were harvested 20 months later and cleaned by ultrasonication in soapy water with a LEO 801 laboratory cleaner (2-L capacity, 80 W, 46 kHz). They were then rinsed with distilled water and assessed by chromatic analysis.

### Choice of colour space

According to Vezhnevets *et al*.^35^, when developing a project that uses colour as the main feature of interest, one usually faces three main issues: the choice of the relevant colour space for the project, the means to obtain and model a colour distribution for your biological model, and the choice of how to process the colour segmentation results in order to obtain a valid and reproducible characterization and quantification of the colour. To reduce the subjectivity of this trait, different colour spaces can be used^55,56^ to shift from qualitative to quantitative data. Among colour space landscapes, the HSL space (Hue Saturation Lightness) is beginning to be used in the biological sciences because it can provide a solution by describing colour components separately^33^. HSV is a colour space similar to HSL and both are used as a convenient way to represent the colour variation. HSL and HSV are both conical geometries, with hue (H) describing the colour spectrum on a chromatic wheel. However, saturation (S) is calculated differently between these two spaces (with a possible conversion between the two values) and lightness (L) and value (V) (referred to here as “brightness” for clearer understanding) represent different aspects of colour. Fairchild^36^ describes brightness (V) as the perception of the amount of light, and the lightness (L) as the perception of the amount of white. V and L are both given in percentages or in [0–1] format. The intuitiveness of this two-colour space and the explicit discrimination between S and L or S and V properties have made these approaches popular in studies on colour segmentation^35^. However, these colour spaces are not perfect. The HSL space, for instance, has an unfortunate interaction between brightness and saturation during image processing. Indeed, for a maximum lightness value, the saturation always gives white data, while this problem does not appear with HSV colour space, which gives a value closer to human vision^34^. We therefore chose the HSV space to analyse shell and pearl chromatic variations resulting from environmental pressure.

### Chromatic analysis of shells and pearls

The shells of the donor oysters and harvested pearls were cleaned, conserved and protected from light. The pearls were put in boxes that classified them by their donor oysters. Both the donors and the boxed pearls were photographed with a Canon® PowerShot G9, with a maximal resolution of 12.1 megapixels and using the same parameters for each picture. Images were taken into a Packshot Creator™ (v. 3.0.3.8) to prevent dark shadows and light reflection. The pictures of the donor’s inner shells were clipped to extract the peripheral coloured zone, which was pasted onto a white background. Similarly, the backgrounds of the pearl pictures were clipped to retain only the coloured sphere and these were pasted onto a white background. We selected one side of each pearl (at random) to be photographed and used this to represent the colour of the pearl. To select the colour area, the free GNU Image Manipulation Program (version 2.8.22) was used (selection with lasso, copy, paste like image, export as.jpeg). R software v 3.2.3 (R foundation for Statistical Computing) was used to develop and run an image analysis package, which we named ImaginR. For the remainder of the analysis, the working directory is set and the folder containing all the pictures is placed inside it. After loading the *ImaginR* (V2) package, the only function needed to run the analysis is called OutPutResult(). Simply by typing OutPutResult(), R will then recognize the picture folder and automatically perform the analysis. *ImaginR* will import all pictures with the.jpeg extension and list the file names into an R object. Thus, the picture’s name (which corresponds to one sample) is stocked into this object. The analysis will therefore be made on a picture-by-picture basis. The picture is imported through the load.image() function of the *ImaginR* package^57^. Then, each pixel is given an RGB (Red Green Blue channels) coding, which will be converted into hex triplet code (all conversions are realized with the *grDevices* package^58^) and compared to a white hex triplet database. The white hex triplet values of the pictures are thus deleted in order to remove any information derived from the background of the picture. The remaining pixels are converted back into an RGB matrix, and an average is calculated for each channel (R, G and B). The average colour of the chromatic zone of each sample is then obtained. The hex triplet code is also calculated from this average. The average RGB is converted into HSV code with the rgb2hsv() function from the *grDevices* package. Thus, for each image, the hue (H), saturation (S) and brightness (V) provide a synthesis of the colour status. The hue variable is compared to a reference hue range that delimits colours to classify the sample into a known phenotype. The reference hue range was delimited with a machine learning procedure using pictures of individuals with the coloured peripheral zones of the two valves extracted as described above. These *ImaginR* database individuals were obtained from a colour breeding selection programme at SCA Regahiga Pearls (Mangareva island, Gambier archipelago, French Polynesia). Approximately 200 individuals of each colour phenotype with a size between 10 and 12 cm were produced. Among these, five individuals per colour phenotype group were selected for their particularly colourful phenotypes and according to colour characteristics sought by pearl farmers. These five individuals per phenotype were then used for the machine learning in order to delimit the phenotype by hue. Following this approach, the hue range for the red phenotype ranged from 0 to 0.1625770; and the hue range for the green phenotype ranged from 0.3215928 to 0.5637775. Finally, the *ImaginR* package provided the hue (H), saturation (S), brightness (V), average hex triplet code and interpreted colour phenotype of each sample (“green”, “red” or “other”) based on the machine learning of that hue. In the procedure, this task is looped over all the samples/pictures in the folder, and the package produces a final tabular file summarizing all the information detailed above, along with the name of the sample. The text file is saved in. csv format. For subsequent statistical analysis, the grouping of the harvested pearl dataset was made in two ways: (i) all the pearls were grouped according to the colour of the donor colour inner shell phenotype characterized in *ImaginR* (since the trait is only partially genetically inherited, pearls with different colour phenotypes were pooled into the same category); (ii) only the green pearls from green donors and red pearls from red donors were analysed.

### Statistical analysis

For the experimental study, we performed several pairwise comparisons to answer two main biological questions: (i) For each colour phenotype, is there a significant difference between the colour of the shells of the donor oysters cultivated at 4 m versus 30 m depth? and (ii) For each phenotype, is there a colour difference between the pearls coming from donors cultivated at 4 m, versus 30 m? A Shapiro test (*stats v3*.*5*.*0* R package) was used to check the normal distribution of the data. To test for the presence of a difference in the brightness and saturation between groups, we used a Wilcoxon test (*stats v3*.*5*.*0* R package based on Hollander and Wolfe^59^ and Patrick Royston^60^) and a confidence interval based on Bauer^61^. Chi^2^ tests were performed (*stats v3*.*5*.*0* R package) on the number of green (or red) pearls obtained from green (or red) oyster donors by donor rearing depth divided by the total number of pearls by rearing depth so as to see which colour phenotype had produced the most pearls of the same colour.

## Data Availability

The authors declare that all data are available.

## References

[1] Williams Suzanne T. (2016). Molluscan shell colour. Biological Reviews.

[2] Bradshaw, A. D. Evolutionary significance of phenotypic plasticity in plants. *Advances in genetics* **13** (Elsevier, 1965).

[3] Moore HB (1936). The Biology of *Purpura Lapillus*. I. Shell Variation in Relation to Environment. J. Mar. Biol. Assoc. United Kingdom.

[4] Manríquez PH, Lagos NA, Jara ME, Castilla JC (2009). Adaptive shell color plasticity during the early ontogeny of an intertidal keystone snail. PNAS.

[5] Kawaii, K. Shell-color polymorphism of intertidal gastropods in Chuuk State, Federated States of Micronesia. *Occas*. *Pap*. 19–22, at http://hdl.handle.net/10232/17204 (2013).

[6] Ding J (2015). Transcriptome sequencing and characterization of Japanese scallop Patinopecten yessoensis from different shell color lines. PLoS One.

[7] Ramirez Boehme, J. New Chilean species of Lucapina, Fissurella and Collisella (Molusca: Archaeogastropoda). *Boletin-Museo Nac*. *Hist*. *Nat*., at http://agris.fao.org/agris-search/search.do?recordID=XL7610034 (1974).

[8] Espoz C, Guzman G, Castilla JC (1995). The lichen *Thelidium litorale* on shells of intertidal limpets: a case of lichen-mediated cryptic mimicry. Mar. Ecol. Prog. Ser..

[9] Williams ST (2017). Colorful seashells: Identification of haem pathway genes associated with the synthesis of porphyrin shell color in marine snails. Ecol. Evol..

[10] Cuthill, I. C. *et al*. The biology of color. *Science (80-*.*)*. **357** (2017). 10.1126/science.aan0221 28774901

[11] Ge J, Li Q, Yu H, Kong L (2015). Mendelian inheritance of golden shell color in the Pacific oyster Crassostrea gigas. Aquaculture.

[12] Feng D, Li Q, Yu H, Zhao X, Kong L (2015). Comparative transcriptome analysis of the pacific oyster Crassostrea gigas characterized by shell colors: Identification of genetic bases potentially involved in pigmentation. PLoS One.

[13] Feng D, Li Q, Yu H, Kong L, Du S (2018). Transcriptional profiling of long non-coding RNAs in mantle of *Crassostrea gigas* and their association with shell pigmentation. Sci. Rep..

[14] Chang YQ (2007). Genetic diversity in five scallop populations of the Japanese scallop (Patinopecten yessoensis). Acta Ecol. Sin..

[15] Evans BS, Knauer J, Taylor JJU, Jerry DR (2006). Development and characterization of six new microsatellite markers for the silver- or gold-lipped pearl oyster, Pinctada maxima (Pteriidae). Mol. Ecol. Notes.

[16] Ky C-L, Lau C, Koua MS, Lo C (2015). Growth Performance Comparison of *Pinctada margaritifera* Juveniles Produced by Thermal Shock or Gonad Scarification Spawning Procedures. J. Shellfish Res..

[17] Blay C (2014). Influence of nacre deposition rate on cultured pearl grade and colour in the black-lipped pearl oyster *Pinctada margaritifera* using farmed donor families. Aquac. Int..

[18] Ky CL (2017). Is pearl colour produced from Pinctada margaritifera predictable through shell phenotypes and rearing environments selections?. Aquac. Res..

[19] Ky Chin-Long, Demmer Jonathan, Blay Carole, Lo Cédrik (2015). Age-dependence of cultured pearl grade and colour in the black-lipped pearl oysterPinctada margaritifera. Aquaculture Research.

[20] Ky C-L (2013). Family effect on cultured pearl quality in black-lipped pearl oyster Pinctada margaritifera and insights for genetic improvement. Aquat. Living Resour..

[21] Ky CL, Lo C, Planes S (2017). Mono- and polychromatic inner shell phenotype diversity in *Pinctada margaritifera* donor pearl oysters and its relation with cultured pearl colour. Aquaculture.

[22] Ky CL, Sham Koua M, Le Moullac G (2018). Impact of spat shell colour selection in hatchery-produced Pinctada margaritifera on cultured pearl colour. Aquac. Reports.

[23] Ky CL, Nakasai S, Molinari N, Devaux D (2014). Influence of grafter skill and season on cultured pearl shape, circles and rejects in *Pinctada margaritifera* aquaculture in Mangareva lagoon. Aquaculture.

[24] Haws, M. *The basic methods of pearl farming: a layman’s manual*, at http://nsgl.gso.uri.edu/hawau/hawauh02001.pdf (Center for Tropical and Subtropical Aquaculture, 2002).

[25] Ky CL, Nakasai S, Pommier S, Sham Koua M, Devaux D (2016). The Mendelian inheritance of rare flesh and shell colour variants in the black-lipped pearl oyster (Pinctada margaritifera). Anim. Genet..

[26] Ky, C.-L., Quillien, V., Broustal, F., Soyez, C. & Devaux, D. Phenome of pearl quality traits in the mollusc transplant model *Pinctada margaritifera*. *Sci*. *Rep*. 1–11, 10.1038/s41598-018-20564-1 (2018). 10.1038/s41598-018-20564-1 PMC5794767 29391512

[27] Cuif J-P (2014). Evidence of a Biological Control over Origin, Growth and End of the Calcite Prisms in the Shells of Pinctada margaritifera (Pelecypod, Pterioidea). Minerals.

[28] Dauphin Y (2008). Structure and composition of the nacre-prisms transition in the shell of *Pinctada margaritifera* (Mollusca, Bivalvia). Anal. Bioanal. Chem..

[29] Marie B, Joubert C, Tayalé A, Zanella-cléon I (2013). Different secretory repertoires control the biomineralization processes of prism and nacre deposition of the pearl oyster shell. Proc. Natl. Acad. Sci. USA.

[30] Snow MR, Pring A, Self P, Losic D, Shapter J (2004). The origin of the color of pearls in iridescence from nano-composite structures of the nacre. Am. Mineral..

[31] Kuriki I (2017). The modern Japanese color lexicon. J. Vis..

[32] Stenger, P.-L. Package ImaginR, at https://cran.r-project.org/web/packages/ImaginR/ImaginR.pdf (2017).

[33] Marchais, V. *et al*. New tool to elucidate the diet of the ormer *Haliotis tuberculata* (L.): Digital shell color analysis. *Mar*. *Biol*. **164** (2017).

[34] Poynton, C. A. *Frequently asked questions about colour*, at http://poynton.ca/PDFs/ColorFAQ.pdf (1995).

[35] Vezhnevets V, Sazonov V, Andreeva A (2003). A Survey on Pixel-Based Skin Color Detection Techniques. Proc. Graph. 2003.

[36] Fairchild, M. D. *Color appearance models* (John Wiley & Sons, 2013).

[37] Trinkler N, Le Moullac G, Cuif J-P, Cochennec-Laureau N, Dauphin Y (2012). Colour or no colour in the juvenile shell of the black lip pearl oyster, Pinctada margaritifera?. Aquat. Living Resour..

[38] Boyle, C. R. & Elston, R. C. Multifactorial genetic models for quantitative traits in humans. *Biometrics* 55–68 (1979). 497338

[39] Bonney GE (1984). On the statistical determination of major gene mechanisms in continuous human traits: Regressive models. Am. J. Med. Genet..

[40] Chandrapavan A, Gardner C, Linnane A, Hobday D (2009). Colour variation in the southern rock lobster *Jasus edwardsii* and its economic impact on the commercial industry. New Zeal. J. Mar. Freshw. Res..

[41] Southgate, P. & Lucas, J. *The pearl oyster* (Elsevier, 2011).

[42] Gervis, M. H. & Sims, N. A. *The biology and culture of pearl oysters (Bivalvia pteriidae)*. **21** (WorldFish, 1992).

[43] Joubert C (2014). Temperature and food influence shell growth and mantle gene expression of shell matrix proteins in the pearl oyster *Pinctada margaritifera*. PLoS One.

[44] Pouvreau, S. Etude et modélisation des mécanismes impliqués dans la croissance de l’huître perlière, *Pinctada margaritifera*, au sein de l’écosystème conchylicole du lagon de l’atoll de Takapoto (Polynésie Française) (1999).

[45] Rousseau M, Rollion-Bard C (2012). Influence of the Depth on the Shape and Thickness of Nacre Tablets of *Pinctada margaritifera* Pearl Oyster, and on Oxygen Isotopic Composition. Minerals.

[46] Latchere O (2017). Influence of preoperative food and temperature conditions on pearl biogenesis in *Pinctada margaritifera*. Aquaculture.

[47] Brown JR, Hartwick EB (1988). Influences of temperature, salinity and available food upon suspended culture of the Pacific oyster, *Crassostrea gigas*. I. Absolute and allometric growth. Aquaculture.

[48] Kanazawa T, Sato S (2008). Environmental and physiological controls on shell microgrowth pattern of *Ruditapes philippinarum* (Bivalvia: Veneridae) from Japan. J. Molluscan Stud..

[49] Thébault J, Thouzeau G, Chauvaud L, Cantillánez M, Avendaño M (2008). Growth of *Argopecten purpuratus* (Mollusca: Bivalvia) on a natural bank in Northern Chile: sclerochronological record and environmental controls. Aquat. Living Resour..

[50] Farre B (2011). Shell layers of the black-lip pearl oyster *Pinctada margaritifera*: Matching microstructure and composition. Comp. Biochem. Physiol. - B Biochem. Mol. Biol..

[51] Jaenisch R, Bird A (2003). Epigenetic regulation of gene expression: How the genome integrates intrinsic and environmental signals. Nat. Genet..

[52] Feil R, Fraga MF (2012). Epigenetics and the environment: Emerging patterns and implications. Nat. Rev. Genet..

[53] Hagmann CA, Schildberg FA, Tolba RH (2010). Epigenetics and transplantation: clinical applications of chromatin regulation. Discov. Med..

[54] McCaughan JA, McKnight AJ, Courtney AE, Maxwell AP (2012). Epigenetics: Time to translate into transplantation. Transplantation.

[55] Clydesdale FM, Ahmed EM (2009). Colorimetry — methodology and applications. Crit. Rev. Food Sci. Nutr..

[56] Hunter, R. S. & Harold, R. W. *The measurement of appearance* (John Wiley & Sons, 1987).

[57] Simon, A., Tschumperle, D. & Wijffels, J. Package imager. 1–126, at https://cran.r-project.org/web/packages/imager/imager.pdf (2018).

[58] R Core Team. grDevices package, at http://www.r-project.org/ (2013).

[59] Hollander, M., A Wolfe, D. & Chicken, E. The one‐way layout. *Nonparametric Stat*. *Methods*, Third Ed. 202–288 (1973).

[60] Royston P (1995). Remark AS R94: A remark on algorithm AS 181: The W-test for normality. J. R. Stat. Soc. Ser. C (Applied Stat..

[61] Bauer DF (1972). Constructing confidence sets using rank statistics. J. Am. Stat. Assoc..

